# Centering, scaling, and transformations: improving the biological information content of metabolomics data

**DOI:** 10.1186/1471-2164-7-142

**Published:** 2006-06-08

**Authors:** Robert A van den Berg, Huub CJ Hoefsloot, Johan A Westerhuis, Age K Smilde, Mariët J van der Werf

**Affiliations:** 1 TNO Quality of Life, P.O. Box 360, 3700 AJ Zeist, The Netherlands; 2 Biosystems Data Analysis, Swammerdam Institute for Life Sciences, Universiteit van Amsterdam, Nieuwe Achtergracht 166, 1018 WV Amsterdam, The Netherlands

## Abstract

**Background:**

Extracting relevant biological information from large data sets is a major challenge in functional genomics research. Different aspects of the data hamper their biological interpretation. For instance, 5000-fold differences in concentration for different metabolites are present in a metabolomics data set, while these differences are not proportional to the biological relevance of these metabolites. However, data analysis methods are not able to make this distinction. Data pretreatment methods can correct for aspects that hinder the biological interpretation of metabolomics data sets by emphasizing the biological information in the data set and thus improving their biological interpretability.

**Results:**

Different data pretreatment methods, i.e. centering, autoscaling, pareto scaling, range scaling, vast scaling, log transformation, and power transformation, were tested on a real-life metabolomics data set. They were found to greatly affect the outcome of the data analysis and thus the rank of the, from a biological point of view, most important metabolites. Furthermore, the stability of the rank, the influence of technical errors on data analysis, and the preference of data analysis methods for selecting highly abundant metabolites were affected by the data pretreatment method used prior to data analysis.

**Conclusion:**

Different pretreatment methods emphasize different aspects of the data and each pretreatment method has its own merits and drawbacks. The choice for a pretreatment method depends on the biological question to be answered, the properties of the data set and the data analysis method selected. For the explorative analysis of the validation data set used in this study, autoscaling and range scaling performed better than the other pretreatment methods. That is, range scaling and autoscaling were able to remove the dependence of the rank of the metabolites on the average concentration and the magnitude of the fold changes and showed biologically sensible results after PCA (principal component analysis).

In conclusion, selecting a proper data pretreatment method is an essential step in the analysis of metabolomics data and greatly affects the metabolites that are identified to be the most important.

## Background

Functional genomics approaches are increasingly being used for the elucidation of complex biological questions with applications that range from human health [1] to microbial strain improvement [2]. Functional genomics tools have in common that they aim to measure the complete biomolecule response of an organism to the environmental conditions of interest. While transcriptomics and proteomics aim to measure all mRNA and proteins, respectively, metabolomics aims to measure all metabolites [3,4].

**Figure 1:**
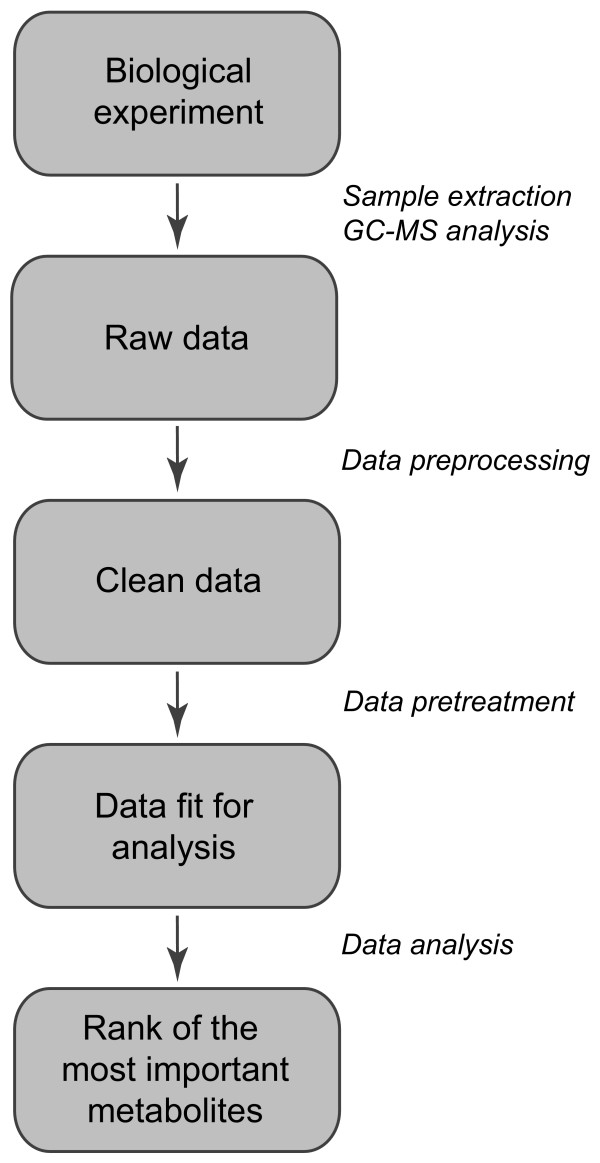
The different steps between biological sampling and ranking of the most important metabolites.

In metabolomics research, there are several steps between the sampling of the biological condition under study and the biological interpretation of the results of the data analysis (Figure 1). First, the biological samples are extracted and prepared for analysis. Subsequently, different data preprocessing steps [3,5] are applied in order to generate 'clean' data in the form of normalized peak areas that reflect the (intracellular) metabolite concentrations. These clean data can be used as the input for data analysis. However, it is important to use an appropriate data pretreatment method before starting data analysis. Data pretreatment methods convert the clean data to a different scale (for instance, relative or logarithmic scale). Hereby, they aim to focus on the relevant (biological) information and to reduce the influence of disturbing factors such as measurement noise. Procedures that can be used for data pretreatment are scaling, centering and transformations.

In this paper, we discuss different properties of metabolomics data, how pretreatment methods influence these properties, and how the effects of the data pretreatment methods can be analyzed. The effect of data pretreatment will be illustrated by the application of eight data pretreatment methods to a metabolomics data set of *Pseudomonas putida *S12 grown on four different carbon sources.

### Properties of metabolome data

In metabolomics experiments, a snapshot of the metabolome is obtained that reflects the cellular state, or phenotype, under the experimental conditions studied [3]. The experiments that resulted in the data set used in this paper were conducted according to an experimental design. In an experimental design, the experimental conditions are purposely chosen to induce variation in the area of interest. The resulting variation in the metabolome is called induced biological variation.

However, other factors are also present in metabolomics data:

1. Differences in orders of magnitude between measured metabolite concentrations; for example, the average concentration of a signal molecule is much lower than the average concentration of a highly abundant compound like ATP. However, from a biological point of view, metabolites present in high concentrations are not necessarily more important than those present at low concentrations.

2. Differences in the fold changes in metabolite concentration due to the induced variation; the concentrations of metabolites in the central metabolism are generally relatively constant, while the concentrations of metabolites that are present in pathways of the secondary metabolism usually show much larger differences in concentration depending on the environmental conditions.

3. Some metabolites show large fluctuations in concentration under identical experimental conditions. This is called uninduced biological variation.

Besides these biological factors, other effects present in the data set are:

4. Technical variation; this originates from, for instance, sampling, sample work-up and analytical errors.

5. Heteroscedasticity; for data analysis, it is often assumed that the total uninduced variation resulting from biology, sampling, and analytical measurements is symmetric around zero with equal standard deviations. However, this assumption is generally not true. For instance, the standard deviation due to uninduced biological variation depends on the average value of the measurement. This is called heteroscedasticity, and it results in the introduction of additional structure in the data [6,7]. Heteroscedasticity occurs in uninduced biological variation as well as in technical variation.

The variation in the data resulting from a metabolomics experiment is the sum of the induced variation and the total uninduced variation. The total uninduced variation is all the variation originating from uninduced biological variation, sampling, sample work-up, and analytical variation. Data pretreatment focuses on the biologically relevant information by emphasizing different aspects in the clean data, for instance, the metabolite concentration under a growth condition relative to the average concentration, or relative to the biological range of that metabolite. In metabolomics, data pretreatment relates the differences in metabolite concentrations in the different samples to differences in the phenotypes of the cells from which these samples were obtained [3].

### Data pretreatment methods

The choice for a data pretreatment method does not only depend on the biological information to be obtained, but also on the data analysis method chosen since different data analysis methods focus on different aspects of the data. For example, a clustering method focuses on the analysis of (dis)similarities, whereas principal component analysis (PCA) attempts to explain as much variation as possible in as few components as possible. Changing data properties using data pretreatment may therefore enhance the results of a clustering method, while obscuring the results of a PCA analysis.

In this paper, we discuss three classes of data pretreatment methods: (I) centering, (II) scaling and (III) transformations (Table 1).

#### Class I: Centering

Centering converts all the concentrations to fluctuations around zero instead of around the mean of the metabolite concentrations. Hereby, it adjusts for differences in the offset between high and low abundant metabolites. It is therefore used to focus on the fluctuating part of the data [8,9], and leaves only the relevant variation (being the variation between the samples) for analysis. Centering is applied in combination with all the methods described below.

#### Class II: Scaling

Scaling methods are data pretreatment approaches that divide each variable by a factor, the scaling factor, which is different for each variable. They aim to adjust for the differences in fold differences between the different metabolites by converting the data into differences in concentration relative to the scaling factor. This often results in the inflation of small values, which can have an undesirable side effect as the influence of the measurement error, that is usually relatively large for small values, is increased as well.

There are two subclasses within scaling. The first class uses a measure of the data dispersion (such as, the standard deviation) as a scaling factor, while the second class uses a size measure (for instance, the mean).

#### Scaling based on data dispersion

Scaling methods tested that use a dispersion measure for scaling were autoscaling [9], pareto scaling [10], range scaling [11], and vast scaling [12] (Table 1). Autoscaling, also called unit or unit variance scaling, is commonly applied and uses the standard deviation as the scaling factor [9]. After autoscaling, all metabolites have a standard deviation of one and therefore the data is analyzed on the basis of correlations instead of covariances, as is the case with centering.

Pareto scaling [10] is very similar to autoscaling. However, instead of the standard deviation, the square root of the standard deviation is used as the scaling factor. Now, large fold changes are decreased more than small fold changes, thus the large fold changes are less dominant compared to clean data. Furthermore, the data does not become dimensionless as after autoscaling (Table 1).

Vast scaling [12] is an acronym of *va*riable *st*ability scaling and it is an extension of autoscaling. It focuses on stable variables, the variables that do not show strong variation, using the standard deviation and the so-called coefficient of variation (cv) as scaling factors (Table 1). The cv is defined as the ratio of the standard deviation and the mean:six¯i
 MathType@MTEF@5@5@+=feaafiart1ev1aaatCvAUfKttLearuWrP9MDH5MBPbIqV92AaeXatLxBI9gBaebbnrfifHhDYfgasaacH8akY=wiFfYdH8Gipec8Eeeu0xXdbba9frFj0=OqFfea0dXdd9vqai=hGuQ8kuc9pgc9s8qqaq=dirpe0xb9q8qiLsFr0=vr0=vr0dc8meaabaqaciaacaGaaeqabaqabeGadaaakeaadaWcaaqaaiabdohaZnaaBaaaleaacqWGPbqAaeqaaaGcbaGafmiEaGNbaebadaWgaaWcbaGaemyAaKgabeaaaaaaaa@32D4@. The use of the cv results in a higher importance for metabolites with a small relative standard deviation and a lower importance for metabolites with a large relative standard deviation. Vast scaling can be used unsupervised as well as supervised. When vast scaling is applied as a supervised method, group information about the samples is used to determine group specific cvs for scaling.

The scaling methods described above use the standard deviation or an associated measure as scaling factor. The standard deviation is, within statistics, a commonly used entity to measure the data spread. In biology, however, a different measure for data spread might be useful as well, namely the biological range. The biological range is the difference between the minimal and the maximal concentration reached by a certain metabolite in a set of experiments. Range scaling [11] uses this biological range as the scaling factor (Table 1). A disadvantage of range scaling with regard to the other scaling methods tested is that only two values are used to estimate the biological range, while for the standard deviation all measurements are taken into account. This makes range scaling more sensitive to outliers. To increase the robustness of range scaling, the range could also be determined by using robust range estimators.

#### Scaling based on average value

Level scaling falls in the second subclass of scaling methods, which use a size measure instead of a spread measure for the scaling. Level scaling converts the changes in metabolite concentrations into changes relative to the average concentration of the metabolite by using the mean concentration as the scaling factor. The resulting values are changes in percentages compared to the mean concentration. As a more robust alternative, the median could be used. Level scaling can be used when large relative changes are of specific biological interest, for example, when stress responses are studied or when aiming to identify relatively abundant biomarkers.

#### Class III: Transformations

Transformations are nonlinear conversions of the data like, for instance, the log transformation and the power transformation (Table 1). Transformations are generally applied to correct for heteroscedasticity [7], to convert multiplicative relations into additive relations, and to make skewed distributions (more) symmetric. In biology, relations between variables are not necessarily additive but can also be multiplicative [13]. A transformation is then necessary to identify such a relation with linear techniques.

Since the log transformation and the power transformation reduce large values in the data set relatively more than the small values, the transformations have a pseudo scaling effect as differences between large and small values in the data are reduced. However, the pseudo scaling effect is not determined by the multiplication with a scaling factor as for a 'real' scaling effect, but by the effect that these transformations have on the original values. This pseudo scaling effect is therefore rarely sufficient to fully adjust for magnitude differences. Hence, it can be useful to apply a scaling method after the transformation. However, it is not clear how the transformation and a scaling method influence each other with regard to the complex metabolomics data.

A transformation that is often used is the log transformation (Table 1). A log transformation perfectly removes heteroscedasticity if the relative standard deviation is constant [7]. However, this is rarely the case in real life situations. A drawback of the log transformation is that it is unable to deal with the value zero. Furthermore, its effect on values with a large relative analytical standard deviation is problematic, usually the metabolites with a relatively low concentration, as these deviations are emphasized. These problems occur because the log transformation approaches minus infinity when the value to be transformed approaches zero.

A transformation that does not show these problems and also has positive effects on heteroscedasticity is the power transformation (Table 1) [13]. The power transformation shows a similar transformation pattern as the log transformation. Hence, the power transformation can be used to obtain results similar as after the log transformation without the near zero artifacts, although the power transformation is not able to make multiplicative effects additive.

## Methods

### Background of the data set

*P. putida *S12 [14] is maintained at TNO. Cultures of *P. putida *S12 were grown in batch fermentations at 30°C in a Bioflow II (New Brunswick Scientific) bioreactor as previously described by van der Werf [15]. Samples (250 ml) were taken from the bioreactor at an OD 600 of 10. Cells were immediately quenched at -45°C in methanol as described previously [16]. Prior to extracting the intracellular metabolites from the cells – by chloroform extraction at -45°C [17] – internal standards were added [18] and a sample was taken for biomass determination [19]. Subsequently, the samples were lyophilized.

### GC-MS analysis

Lyophilized metabolome samples were derivatized using a solution of ethoxyamine hydrochloride in pyridine as the oximation reagent followed by silylation with N-trimethyl-N-trimethylsilylacetamide as described by [18]. GC-MS-analysis of the derivatized samples was performed using temperature gradient from 70°C to 320°C at a rate of 10°C/min on an Agilent 6890 N GC (Palo Alto, CA, USA) and an Agilent 5973 mass selective detector. 1 μl aliquots of the derivatized samples were injected in the splitless mode on a DB5-MS capillary column. Detection was performed using MS detection in electron impact mode (70 eV).

### Data preprocessing

**Figure 2:**
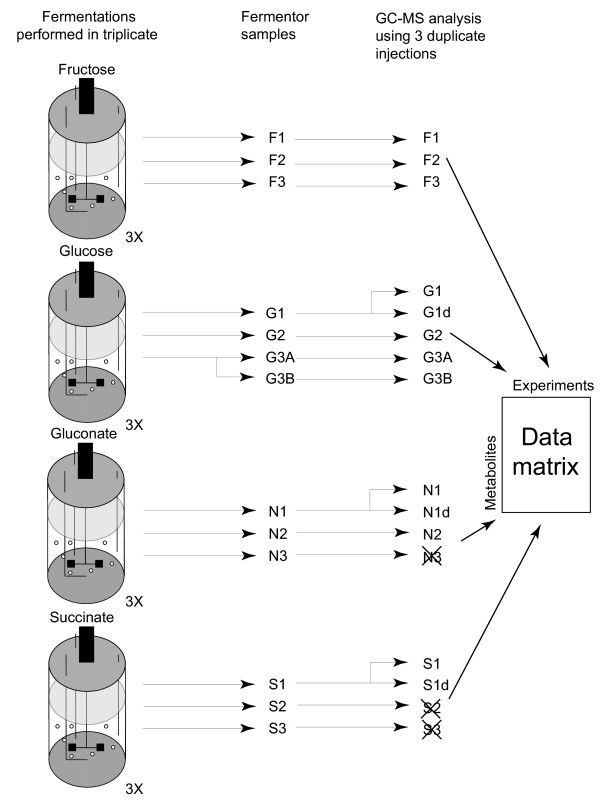
**Experimental design**. The fermentations were performed in independent triplicates. Of the third glucose fermentation a sample was taken in duplicate and of G1, N1 and S1 the samples were analyzed in duplicate by GC-MS. The samples of N3, S2 and S3 were not taken into account in this study.

The data from GC-MS analyses were deconvoluted using the AMDIS spectral deconvolution software package [18,20]. Zeros in the data set were replaced with small values equal to MS peak areas of 1 to allow for log transformations. The lowest peak areas in the rest of the data are in the order of 10^3^. The output of the AMDIS analysis, in the form of peak identifiers and peak areas, was corrected for the recovery of internal standards and normalized with respect to biomass. The peaks resulting from a known compound were combined. The samples N3, S2 and S3 were removed from the data set, as a different sample workup protocol was followed. Furthermore, metabolites detected only once in the 13 remaining experiments were removed. This lead to a reduced data set consisting of 13 experiments and 140 variables expressed as peak areas in arbitrary units (Figure 2). This data set was used as the clean data for data pretreatment.

### Data pretreatment

Data pretreatment and PCA were performed using Matlab 7 [21], the PLS Toolbox 3.0 [22], and home written m-files. Data pretreatment was applied according to the formulas in Table 1. The notation of the formulas is as follows: Matrices are presented in bold uppercase (**X**), vectors in bold lowercase (**t**), and scalars are given in lowercase italic (*a*) or uppercase italic in case of the end of a series *i *= 1...*I*. The data is presented in a data matrix **X **(*I *× *J*) with *I *rows referring to the metabolites and *J *columns referring to the different conditions. Element *x*_*ij *_therefore holds the measurement of metabolite *i *in experiment *j*.

Vast scaling was applied unsupervised as the other data pretreatment methods were unsupervised as well.

### Data analysis

PCA was applied for the analysis of the data. PCA decomposes the variation of matrix **X **into scores **T**, loadings **P**, and a residuals matrix **E**. **P **is an *I *× *A *matrix containing the *A *selected loadings and **T **is a *J *× *A *matrix containing the accompanying scores.

**X **= **PT**^*T *^+ **E**,

where ***P***^*T *^***P ***= ***I***, the identity matrix.

The number of components used (*A*) in the PCA analysis was based on the scree plots and the score plots.

For ranking of the metabolites according to importance for the *A *selected PCs, the contribution *r *of all the variables to the effects observed in the *A *PCs was calculated

rAi=∑a=1Aλa2⋅pia2
 MathType@MTEF@5@5@+=feaafiart1ev1aaatCvAUfKttLearuWrP9MDH5MBPbIqV92AaeXatLxBI9gBaebbnrfifHhDYfgasaacH8akY=wiFfYdH8Gipec8Eeeu0xXdbba9frFj0=OqFfea0dXdd9vqai=hGuQ8kuc9pgc9s8qqaq=dirpe0xb9q8qiLsFr0=vr0=vr0dc8meaabaqaciaacaGaaeqabaqabeGadaaakeaacqWGYbGCdaWgaaWcbaGaemyqaeKaemyAaKgabeaakiabg2da9maaqahabaGaeq4UdW2aa0baaSqaaiabdggaHbqaaiabikdaYaaakiabgwSixlabdchaWnaaDaaaleaacqWGPbqAcqWGHbqyaeaacqaIYaGmaaaabaGaemyyaeMaeyypa0JaeGymaedabaGaemyqaeeaniabggHiLdaaaa@43DE@

Here, *r *is the contribution of variable *i *to *A *components, λ_*a *_is the singular value for the *a*^th ^PC and *p*_*ia *_is the value for the *i*^th ^variable in the loading vector belonging to the *a*^th ^PC. To allow for comparison between the different data pretreatment methods, the values for *r*_*A *_were sorted in descending order after which the comparisons were performed using the rank of the metabolite in the sorted list.

The measurement errors were analyzed by estimation of the standard deviation from the biological, analytical, and sampling repeats. The standard deviations were binned by calculating the average variance per 10 metabolites ordered by mean value [23].

The jackknife routine was performed according to the following setup. In round one experiments F1, G1, N1 were left out, in round two F2, G2, N1d were left out, and in round three F3, G3A, were left out. By selecting these experiments, the specific aspects of the experimental design were maintained.

## Results and discussion

### Properties of the clean data

For any data set, the total variation is the sum of the contributions of all the different sources of variation. The sources of variation in the data set used in this study were the induced biological variation, the uninduced biological variation, the sample work-up variation, and the analytical variation. The variation resulting from the sample work-up and the analytical analysis together was called technical variation. The contributions of the different sources of variation were roughly estimated from the replicate measurements by calculating the sum of squares (SS) and the mean square (MS) (Table 2). In this data set, the largest contribution to the variation originated from the induced biological variation, followed by the uninduced biological variation. The analytical variation was the smallest source of variation (Table 2).

### The effect of pretreatment on the clean data

**Figure 3:**
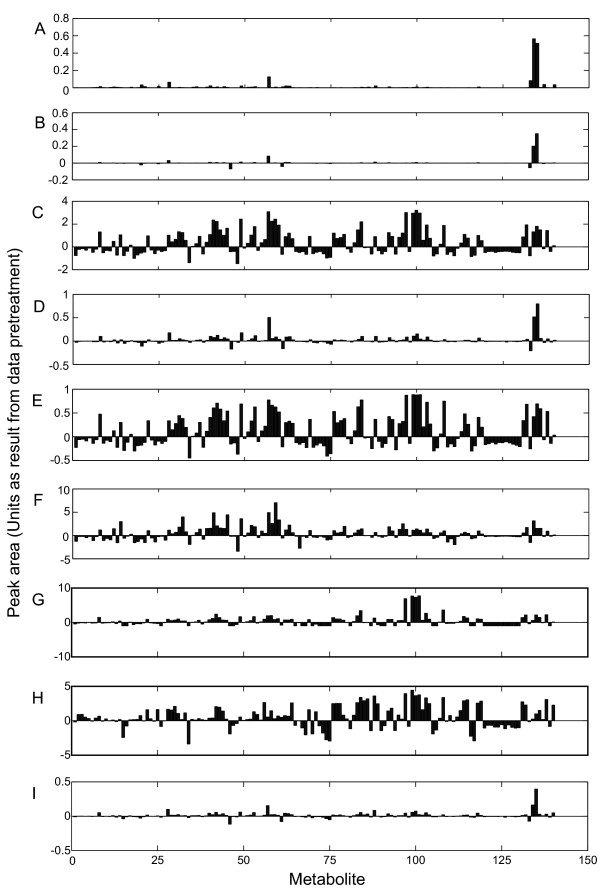
**Effect of data pretreatment on the original data**. Original data of experiment G2 (A), and the data after centering (B), autoscaling (C), pareto scaling (D), range scaling (E), vast scaling (F), level scaling (G), log transformation (H), and power transformation (I). For units refer to Table 1.

The application of different pretreatment methods on the clean data had a large effect on the resulting data used as input for data analysis, as is depicted for sample G2 in Figure 3. The different pretreatment methods resulted in different effects. For instance autoscaling (Figure 3C) showed many large peaks, while after pareto scaling (Figure 3D), only a few large peaks were present. It is evident that different results will be obtained when the in different ways pretreated data sets are used as the input for data analysis.

### Heteroscedasticity

**Figure 4:**
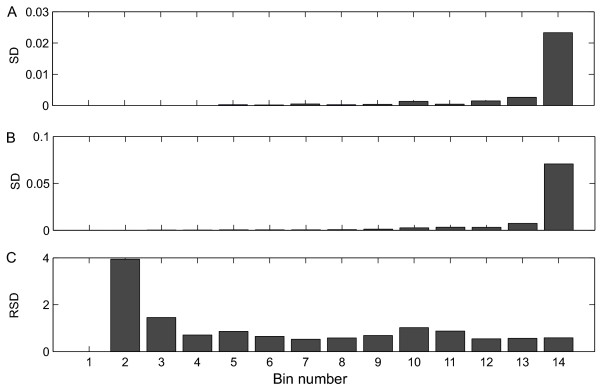
**Analytical and biological heteroscedasticity in the data**. A: Analytical standard deviation (experiment G1), B: Biological standard deviation (all glucose experiments), and C: Relative biological standard deviation (all glucose experiments), as a function of the metabolite concentration. To obtain a clearer overview, the standard deviations were grouped together based on average mean value of the peak area (Binning, see Jansen *et al*. [23]). The first bin contained the metabolites whose peak area was below the detection limit.

To determine the presence or absence of heteroscedasticity in the data set, the standard deviations of the metabolites of the analytical and the biological repeats were analyzed (Figure 4). Analysis of the analytical and the uninduced biological standard deviations showed that heteroscedasticity was present both in the analytical error and in the biological uninduced variation (Figure 4A and 4B). In contrast, the *relative *biological standard deviation (Figure 4C), and also the relative analytical standard deviation (unpublished results), showed the opposite effect. Thus, metabolites present in high concentrations were relatively influenced less by the disturbances resulting from the different sources of uninduced variation, and were therefore more reliable.

**Figure 5:**
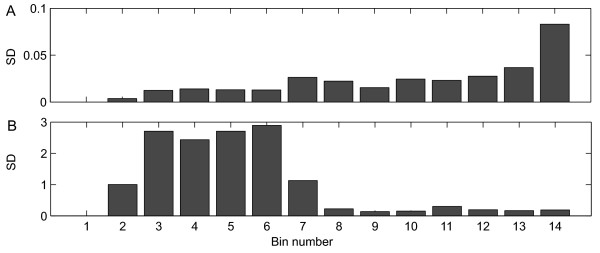
**Effect of data transformation on biological heteroscedasticity**. A: power transformed data. B: log transformed data. The standard deviations over all glucose experiments were ordered by the mean value of the peak areas and binned per 10 metabolites. The first bin contained the metabolites whose peak area was below the detection limit.

The effect of the log and the power transformation on the data as a means to correct for heteroscedasticity is shown in Figure 5. Compared to the clean data (Figure 4B), the heteroscedasticity was reduced by the power transformation (Figure 5A), although the power transformation was not able to remove it completely. The results can possibly be improved further if a different power would be used (Box and Cox [24]). Also, the log transformation (Figure 5B) was able to remove heteroscedasticity, however only for the metabolites that are present in high concentrations. In contrast, the standard deviations of metabolites present in low concentrations were inflated after log transformation due to the large relative standard deviation of these low abundant metabolites.

Scaling approaches influence the heteroscedasticity as well, since the variation, and thus the heteroscedasticity, is converted into relative values to the scaling factor. It is likely that this aspect reduces the effect of the heteroscedasticity on the results.

### The effect of data pretreatment on the data analysis results

PCA [9,25] was applied to analyze the effect on the data analysis for the in different ways pretreated data. PCA was chosen as it is an explorative tool that is able to visualize how the data pretreatment methods are able to reveal different aspects of the data in the scores and the accompanying loadings. Furthermore, it allows for identification of the most important metabolites for the biological problem by analysis of the loadings.

The score plots were judged on two aspects by visual inspection, namely the distance within the cluster of a specific carbon source and the distance between the clusters of different carbon sources. The loading plots show the contributions of the measured metabolites to the separation of the experiments in the score plots. As cellular metabolism is strongly interlinked (e.g. see [26,27]), it is expected that the concentrations of many metabolites are simultaneously affected when an organism is grown on a different carbon source. Therefore, the loadings are expected to show contributions of many different metabolites.

**Figure 6:**
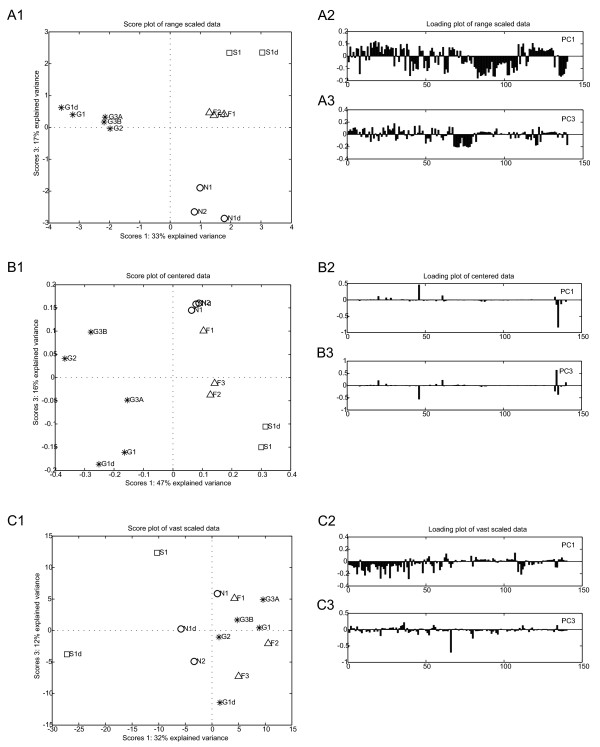
**Effect of data pretreatment on the PCA results**. PCA results of range scaled data (6A), centered data (6B), and vast scaled data (6C). For every pretreatment method the score plot (X1) (PC1 vs. PC2) and the loadings of PC 1 (X2) and PC 2 (X3) are shown. D-fructose (F, △), succinate (S, □), D-gluconate (N, ◯), D-glucose (G, *).

The data pretreatment methods used largely affected the outcome of PCA analysis (Figure 6). Three groups of data pretreatment methods could be identified in this way. After range scaling, a clear clustering of the samples was observed based on the carbon sources on which the sampled cells were grown (Figure 6A1). Furthermore, the loading plots (Figure 6A2 and 6A3) indicate that many metabolites contributed to the effects in the score plots; which is in agreement with the biological expectation. Autoscaling, level scaling, and log transformation resulted in similar PCA results as after range scaling (unpublished results).

The application of centering lead to intermediate clustering results in the score plots (Figure 6B1). The clusters were larger and less well separated compared to the results for range scaling (Figure 6A1). The most striking results for centered data are visible in the loading plots (Figure 6B2 and 6B3). Only a few metabolites had very large contributions to the effects shown the score plot (Figure 6B1), which is in disagreement with the biological expectations. Power transformation and pareto scaling gave similar PCA results (unpublished results).

In contrast to the other pretreatment methods, vast scaling of the clean data resulted in a very poor clustering of the samples (Figure 6C1). Overlapping clusters were observed, although the loading plots (Figure 6C2 and 6C3) show contributions of many metabolites.

These results clearly demonstrate that the pretreatment method chosen dramatically influences the results of a PCA analysis. Consequently, these effects are also present in the rank of the metabolites.

### Ranking of the most important metabolites

**Figure 7:**
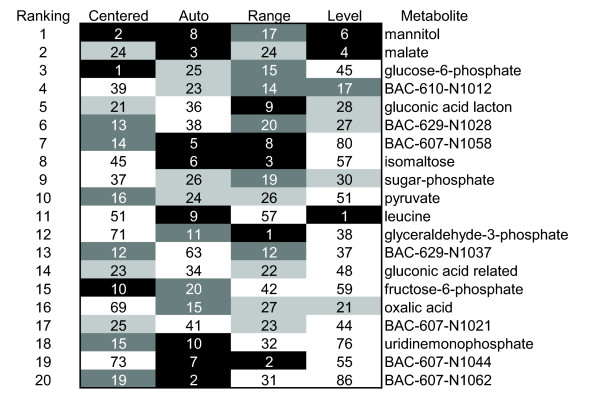
**Rank of the most important metabolites**. The rank was based on the cumulative contributions of the loadings of the first three PCs. Top 10 metabolites are given in white characters with a black background, the top 11 to 20 is given in white characters with dark gray background, the top 21 to 30 is given in black characters with a light gray background.

In functional genomics research, ranking of targets according to their relevance to the problem studied (for instance, strain improvement) is of great importance as it is time consuming and costly to validate the, in general, dozens or hundreds of leads that are generated in these studies[2]. As shown in Figure 6, the use of different pretreatment methods influenced the PCA analysis and the resulting loadings. For the different pretreatment methods, different metabolites were identified as the most important by studying the cumulative contributions of the loadings of the metabolites on PCs 1, 2 and 3 (Figure 7). Glucose-6-phosphate, for instance, was identified as the most important metabolite when using centering as the pretreatment method, while glyceraldehyde-3-phosphate (GAP) was identified as the most important metabolite when applying range scaling. For centering, autoscaling, and level scaling, GAP was the 71^st^, 11^th^, or 38^th ^most important metabolite, respectively. The pretreatment of the clean data thus directly affected the ranking of the metabolites as being the most relevant.

**Figure 8:**
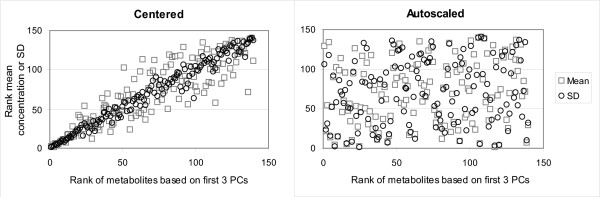
**Relation between the abundance or the fold change of a metabolite and its rank after data pretreatment**. The highest ranked metabolite after data pretreatment, based on its cumulative contributions on the loadings of the first three PCs, has position 1 on the X-axis. The metabolite that is ranked at position 1 on the Y-axis has either the highest fold change in concentration (largest standard deviation of the peak area over all the experiments in the clean data (O)); or is most abundant (largest mean concentration (□)) in the clean data.

The effect of a data pretreatment method on the rank of the metabolites is also apparent when studying the relation between the rank of the metabolites and the abundance (average peak area of a metabolite), or the fold change (standard deviation of the peak area over all experiments for a metabolite) (Figure 8). The effect of autoscaling (Figure 8B), and also range scaling (unpublished results), is in agreement with the expectation that the average concentration and the magnitude of the fold change are not a measure for the biological relevance of a metabolite. In contrast, with centering (Figure 8A), pareto scaling, level scaling, log transformation, and power transformation (unpublished results), a clear relation between the rank of the metabolites and the abundance, or the fold change, of a metabolite was observed. This relation was less obvious for vast scaling, however still present (unpublished results).

### Reliability of the rank of the metabolites

While the rank of the metabolites provides valuable information, the robustness of this rank is just as important as it determines the limits of the reliable interpretation of the rank. To test the reliability of the rank of the metabolites, a jackknife routine was applied [28].

**Figure 9:**
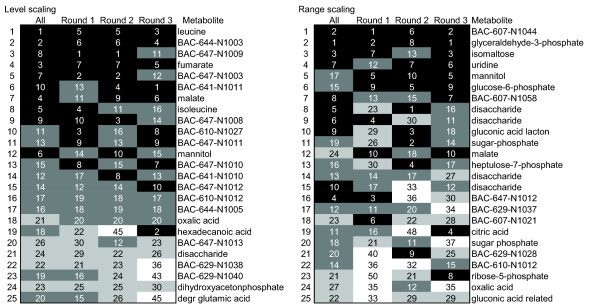
**Stability of the rank of the most important metabolites**. The order of the metabolites is based on the average rank.

The results for level scaling and range scaling are shown in Figure 9. The highest ranking metabolites (up to the eighth position) for both level scaled and range scaled data were relatively stable. For both methods, the fluctuations became larger for lower ranked metabolites, however, for the rank based on range scaled data the fluctuations in the rank increased faster than for the data resulting from level scaled data.

This resampling approach showed that the reliability of the rank of the most important metabolites is also dependent on the data pretreatment method. The most stable data pretreatment methods were centering, level scaling (Figure 9), log transformation, power transformation, pareto scaling, and vast scaling (results not shown). Autoscaling was less stable (results not shown), while the least stable data pretreatment method was range scaling. Two factors affect the reliability of the rank of the metabolites. The first factor relates to the reliability with which the scaling factor can be determined. For instance, level scaling uses the mean as the scaling factor. As the mean is based on all the measurements, it is quite stable. On the other hand, range scaling uses the biological range observed in the data as a scaling factor, which is based on two values only. The second factor that influences the reliability of the rank relates to those data pretreatment methods whose subsequent data analysis results show a preference for the high abundant metabolites (Figure 8). With these pretreatment methods, the stability of the rank is predetermined by this character due to the low relative standard deviation of the uninduced biological variation of the high abundant metabolites (Figure 4B).

It must be stressed that the pretreatment method that provides the most stable rank does not necessarily provides the most relevant biological answers.

## Conclusion

This paper demonstrates that the data pretreatment method used is crucial to the outcome of the data analysis of functional genomics data. The selection of a data pretreatment method depends on three factors: (i) the biological question that has to be answered, (ii) the properties of the data set, and (iii) the data analysis method that will be used for the analysis of the functional genomics data.

Notwithstanding these boundaries, autoscaling and range scaling seem to perform better than the other methods with regard to the biological expectations. That is, range scaling and autoscaling were able to remove the dependence of the rank of the metabolites on the average concentration and the magnitude of the fold changes and showed biologically sensible results after PCA analysis. Other methods showed a strong dependence on the average concentration or magnitude of the fold change (centering, log transformation, power transformation, level scaling, pareto scaling), or lead to PCA results that were poorly interpretable in relation to the experimental setup (vast scaling).

Using a pretreatment method that is not suited for the biological question, the data, or the data analysis method, will lead to poor results with regard to, for instance, the rank of the most relevant metabolites for the biological question that is subject of study (Figure 7 and 8). This will therefore result in a wrong biological interpretation of the results.

In functional genomics data analysis, data pretreatment is often overlooked or is applied in an ad hoc way. For instance, in many software packages, such as Cluster [29] and the PLS toolbox [22], data pretreatment is integrated in the data analysis program and can be easily turned on or off. This can lead to a careless search through different pretreatment methods until the results best fit the expectations of the researcher. Therefore, we advise against method mining. With method mining, the best result translates to 'which method fits the expectations the best'. This is poor practice, as results cannot be considered reliable when the assumptions and limitations of a data pretreatment method are not taken into account. Furthermore, it is sometimes unknown what to expect, or the starting hypothesis is incorrect.

As far as we are aware, this is the first time that the importance of selecting a proper data pretreatment method on the outcome of data analysis in relation to the identification of biologically important metabolites in metabolomics/functional genomics is clearly demonstrated.

## Authors' contributions

RAB is responsible for the idea of a comprehensive comparison of data pretreatment methods, and performed the statistical analyses. HCJH provided valuable input with regard to the mathematical soundness of the research. JAW advised on the practical issues and the interpretation of the results. AKS supplied statistical feedback and conceptual feedback on different pretreatment methods. MJW recognized the importance of data pretreatment for biological interpretation and kept the focus of the research on biological interpretability.

## Figures and Tables

**Table 1:** Overview of the pretreatment methods used in this study. In the Unit column, the unit of the data after the data pretreatment is stated. *O *represents the original Unit, and (-) presents dimensionless data. The mean is estimated as: x¯i=1J∑j=1Jxij
 MathType@MTEF@5@5@+=feaafiart1ev1aaatCvAUfKttLearuWrP9MDH5MBPbIqV92AaeXatLxBI9gBaebbnrfifHhDYfgasaacH8akY=wiFfYdH8Gipec8Eeeu0xXdbba9frFj0=OqFfea0dXdd9vqai=hGuQ8kuc9pgc9s8qqaq=dirpe0xb9q8qiLsFr0=vr0=vr0dc8meaabaqaciaacaGaaeqabaqabeGadaaakeaacuWG4baEgaqeamaaBaaaleaacqWGPbqAaeqaaOGaeyypa0ZaaSaaaeaacqaIXaqmaeaacqWGkbGsaaWaaabCaeaacqWG4baEdaWgaaWcbaGaemyAaKMaemOAaOgabeaaaeaacqWGQbGAcqGH9aqpcqaIXaqmaeaacqWGkbGsa0GaeyyeIuoaaaa@3DF5@ and the standard deviation is estimated as: si=∑j=1J(xij−x¯i)2J−1
 MathType@MTEF@5@5@+=feaafiart1ev1aaatCvAUfKttLearuWrP9MDH5MBPbIqV92AaeXatLxBI9gBaebbnrfifHhDYfgasaacH8akY=wiFfYdH8Gipec8Eeeu0xXdbba9frFj0=OqFfea0dXdd9vqai=hGuQ8kuc9pgc9s8qqaq=dirpe0xb9q8qiLsFr0=vr0=vr0dc8meaabaqaciaacaGaaeqabaqabeGadaaakeaacqWGZbWCdaWgaaWcbaGaemyAaKgabeaakiabg2da9maakaaabaWaaSaaaeaadaaeWbqaamaabmaabaGaemiEaG3aaSbaaSqaaiabdMgaPjabdQgaQbqabaGccqGHsislcuWG4baEgaqeamaaBaaaleaacqWGPbqAaeqaaaGccaGLOaGaayzkaaWaaWbaaSqabeaacqaIYaGmaaaabaGaemOAaOMaeyypa0JaeGymaedabaGaemOsaOeaniabggHiLdaakeaacqWGkbGscqGHsislcqaIXaqmaaaaleqaaaaa@45A6@. x˜
 MathType@MTEF@5@5@+=feaafiart1ev1aaatCvAUfKttLearuWrP9MDH5MBPbIqV92AaeXatLxBI9gBaebbnrfifHhDYfgasaacH8akY=wiFfYdH8Gipec8Eeeu0xXdbba9frFj0=OqFfea0dXdd9vqai=hGuQ8kuc9pgc9s8qqaq=dirpe0xb9q8qiLsFr0=vr0=vr0dc8meaabaqaciaacaGaaeqabaqabeGadaaakeaacuWG4baEgaacaaaa@2E34@ and x^
 MathType@MTEF@5@5@+=feaafiart1ev1aaatCvAUfKttLearuWrP9MDH5MBPbIqV92AaeXatLxBI9gBaebbnrfifHhDYfgasaacH8akY=wiFfYdH8Gipec8Eeeu0xXdbba9frFj0=OqFfea0dXdd9vqai=hGuQ8kuc9pgc9s8qqaq=dirpe0xb9q8qiLsFr0=vr0=vr0dc8meaabaqaciaacaGaaeqabaqabeGadaaakeaacuWG4baEgaqcaaaa@2E35@ represent the data after different pretreatment steps.

**Class**	**Method**	**Formula**	**Unit**	**Goal**	**Advantages**	**Disadvantages**
**I**	Centering	x˜ij=xij−x¯i MathType@MTEF@5@5@+=feaafiart1ev1aaatCvAUfKttLearuWrP9MDH5MBPbIqV92AaeXatLxBI9gBaebbnrfifHhDYfgasaacH8akY=wiFfYdH8Gipec8Eeeu0xXdbba9frFj0=OqFfea0dXdd9vqai=hGuQ8kuc9pgc9s8qqaq=dirpe0xb9q8qiLsFr0=vr0=vr0dc8meaabaqaciaacaGaaeqabaqabeGadaaakeaacuWG4baEgaacamaaBaaaleaacqWGPbqAcqWGQbGAaeqaaOGaeyypa0JaemiEaG3aaSbaaSqaaiabdMgaPjabdQgaQbqabaGccqGHsislcuWG4baEgaqeamaaBaaaleaacqWGPbqAaeqaaaaa@3A94@	*O*	Focus on the differences and not the similarities in the data	Remove the offset from the data	When data is heteroscedastic, the effect of this pretreatment method is not always sufficient

**II**	Autoscaling	x˜ij=xij−x¯isi MathType@MTEF@5@5@+=feaafiart1ev1aaatCvAUfKttLearuWrP9MDH5MBPbIqV92AaeXatLxBI9gBaebbnrfifHhDYfgasaacH8akY=wiFfYdH8Gipec8Eeeu0xXdbba9frFj0=OqFfea0dXdd9vqai=hGuQ8kuc9pgc9s8qqaq=dirpe0xb9q8qiLsFr0=vr0=vr0dc8meaabaqaciaacaGaaeqabaqabeGadaaakeaacuWG4baEgaacamaaBaaaleaacqWGPbqAcqWGQbGAaeqaaOGaeyypa0ZaaSaaaeaacqWG4baEdaWgaaWcbaGaemyAaKMaemOAaOgabeaakiabgkHiTiqbdIha4zaaraWaaSbaaSqaaiabdMgaPbqabaaakeaacqWGZbWCdaWgaaWcbaGaemyAaKgabeaaaaaaaa@3DA4@	(-)	Compare metabolites based on correlations	All metabolites become equally important	Inflation of the measurement errors
	Range scaling	x˜ij=xij−x¯i(ximax⁡−ximin⁡) MathType@MTEF@5@5@+=feaafiart1ev1aaatCvAUfKttLearuWrP9MDH5MBPbIqV92AaeXatLxBI9gBaebbnrfifHhDYfgasaacH8akY=wiFfYdH8Gipec8Eeeu0xXdbba9frFj0=OqFfea0dXdd9vqai=hGuQ8kuc9pgc9s8qqaq=dirpe0xb9q8qiLsFr0=vr0=vr0dc8meaabaqaciaacaGaaeqabaqabeGadaaakeaacuWG4baEgaacamaaBaaaleaacqWGPbqAcqWGQbGAaeqaaOGaeyypa0ZaaSaaaeaacqWG4baEdaWgaaWcbaGaemyAaKMaemOAaOgabeaakiabgkHiTiqbdIha4zaaraWaaSbaaSqaaiabdMgaPbqabaaakeaadaqadaqaaiabdIha4naaBaaaleaacqWGPbqAdaWgaaadbaGagiyBa0MaeiyyaeMaeiiEaGhabeaaaSqabaGccqGHsislcqWG4baEdaWgaaWcbaGaemyAaK2aaSbaaWqaaiGbc2gaTjabcMgaPjabc6gaUbqabaaaleqaaaGccaGLOaGaayzkaaaaaaaa@4BF0@	(-)	Compare metabolites relative to the biological response range	All metabolites become equally important. Scaling is related to biology	Inflation of the measurement errors and sensitive to outliers
	Pareto scaling	x˜ij=xij−x¯isi MathType@MTEF@5@5@+=feaafiart1ev1aaatCvAUfKttLearuWrP9MDH5MBPbIqV92AaeXatLxBI9gBaebbnrfifHhDYfgasaacH8akY=wiFfYdH8Gipec8Eeeu0xXdbba9frFj0=OqFfea0dXdd9vqai=hGuQ8kuc9pgc9s8qqaq=dirpe0xb9q8qiLsFr0=vr0=vr0dc8meaabaqaciaacaGaaeqabaqabeGadaaakeaacuWG4baEgaacamaaBaaaleaacqWGPbqAcqWGQbGAaeqaaOGaeyypa0ZaaSaaaeaacqWG4baEdaWgaaWcbaGaemyAaKMaemOAaOgabeaakiabgkHiTiqbdIha4zaaraWaaSbaaSqaaiabdMgaPbqabaaakeaadaGcaaqaaiabdohaZnaaBaaaleaacqWGPbqAaeqaaaqabaaaaaaa@3DB4@	*O*	Reduce the relative importance of large values, but keep data structure partially intact	Stays closer to the original measurement than autoscaling	Sensitive to large fold changes
	Vast scaling	x˜ij=(xij−x¯i)si⋅x¯isi MathType@MTEF@5@5@+=feaafiart1ev1aaatCvAUfKttLearuWrP9MDH5MBPbIqV92AaeXatLxBI9gBaebbnrfifHhDYfgasaacH8akY=wiFfYdH8Gipec8Eeeu0xXdbba9frFj0=OqFfea0dXdd9vqai=hGuQ8kuc9pgc9s8qqaq=dirpe0xb9q8qiLsFr0=vr0=vr0dc8meaabaqaciaacaGaaeqabaqabeGadaaakeaacuWG4baEgaacamaaBaaaleaacqWGPbqAcqWGQbGAaeqaaOGaeyypa0ZaaSaaaeaadaqadaqaaiabdIha4naaBaaaleaacqWGPbqAcqWGQbGAaeqaaOGaeyOeI0IafmiEaGNbaebadaWgaaWcbaGaemyAaKgabeaaaOGaayjkaiaawMcaaaqaaiabdohaZnaaBaaaleaacqWGPbqAaeqaaaaakiabgwSixpaalaaabaGafmiEaGNbaebadaWgaaWcbaGaemyAaKgabeaaaOqaaiabdohaZnaaBaaaleaacqWGPbqAaeqaaaaaaaa@47A9@	(-)	Focus on the metabolites that show small fluctuations	Aims for robustness, can use prior group knowledge	Not suited for large induced variation without group structure
	Level scaling	x˜ij=xij−x¯ix¯i MathType@MTEF@5@5@+=feaafiart1ev1aaatCvAUfKttLearuWrP9MDH5MBPbIqV92AaeXatLxBI9gBaebbnrfifHhDYfgasaacH8akY=wiFfYdH8Gipec8Eeeu0xXdbba9frFj0=OqFfea0dXdd9vqai=hGuQ8kuc9pgc9s8qqaq=dirpe0xb9q8qiLsFr0=vr0=vr0dc8meaabaqaciaacaGaaeqabaqabeGadaaakeaacuWG4baEgaacamaaBaaaleaacqWGPbqAcqWGQbGAaeqaaOGaeyypa0ZaaSaaaeaacqWG4baEdaWgaaWcbaGaemyAaKMaemOAaOgabeaakiabgkHiTiqbdIha4zaaraWaaSbaaSqaaiabdMgaPbqabaaakeaacuWG4baEgaqeamaaBaaaleaacqWGPbqAaeqaaaaaaaa@3DC6@	(-)	Focus on relative response	Suited for identification of e.g. biomarkers	Inflation of the measurement errors

**III**	Log transformation	x˜ij=10log⁡(xij)x⌢ij=x˜ij−x˜¯i MathType@MTEF@5@5@+=feaafiart1ev1aaatCvAUfKttLearuWrP9MDH5MBPbIqV92AaeXatLxBI9gBaebbnrfifHhDYfgasaacH8akY=wiFfYdH8Gipec8Eeeu0xXdbba9frFj0=OqFfea0dXdd9vqai=hGuQ8kuc9pgc9s8qqaq=dirpe0xb9q8qiLsFr0=vr0=vr0dc8meaabaqaciaacaGaaeqabaqabeGadaaakqaabeqaaiqbdIha4zaaiaWaaSbaaSqaaiabdMgaPjabdQgaQbqabaGccqGH9aqpdaahaaWcbeqaaiabigdaXiabicdaWaaakiGbcYgaSjabc+gaVjabcEgaNnaabmaabaGaemiEaG3aaSbaaSqaaiabdMgaPjabdQgaQbqabaaakiaawIcacaGLPaaaaeaacuWG4baEgaWeamaaBaaaleaacqWGPbqAcqWGQbGAaeqaaOGaeyypa0JafmiEaGNbaGaadaWgaaWcbaGaemyAaKMaemOAaOgabeaakiabgkHiTiqbdIha4zaaiyaaraWaaSbaaSqaaiabdMgaPbqabaaaaaa@4C62@	Log *O*	Correct for heteroscedasticity, pseudo scaling. Make multiplicative models additive	Reduce heteroscedasticity, multiplicative effects become additive	Difficulties with values with large relative standard deviation and zeros
	Power transformation	x˜ij=(xij)x⌢ij=x˜ij−x˜¯i MathType@MTEF@5@5@+=feaafiart1ev1aaatCvAUfKttLearuWrP9MDH5MBPbIqV92AaeXatLxBI9gBaebbnrfifHhDYfgasaacH8akY=wiFfYdH8Gipec8Eeeu0xXdbba9frFj0=OqFfea0dXdd9vqai=hGuQ8kuc9pgc9s8qqaq=dirpe0xb9q8qiLsFr0=vr0=vr0dc8meaabaqaciaacaGaaeqabaqabeGadaaakqaabeqaaiqbdIha4zaaiaWaaSbaaSqaaiabdMgaPjabdQgaQbqabaGccqGH9aqpdaGcaaqaamaabmaabaGaemiEaG3aaSbaaSqaaiabdMgaPjabdQgaQbqabaaakiaawIcacaGLPaaaaSqabaaakeaacuWG4baEgaWeamaaBaaaleaacqWGPbqAcqWGQbGAaeqaaOGaeyypa0JafmiEaGNbaGaadaWgaaWcbaGaemyAaKMaemOAaOgabeaakiabgkHiTiqbdIha4zaaiyaaraWaaSbaaSqaaiabdMgaPbqabaaaaaa@4654@	*√O*	Correct for heteroscedasticity, pseudo scaling	Reduce heteroscedasticity, no problems with small values	Choice for square root is arbitrary.

**Table 2:** Estimation of the sources of variation in the data set. The SS and the MS for the different sources of variation are given, based on the experimental design presented in Figure 2. *The technical source of variation consists of the analytical error and the sample work-up error.

**Source of variation**	**SS**	**MS**
Analytical	0.0205	0.0102
Technical*	0.0482	0.0482
Uninduced biological	0.208	0.104
Induced biological	0.952	0.317
	
Total SS	1.23	
